# Annual U.S. healthcare expenditures attributable to cigar smoking between 2001 and 2018, overall and by payer

**DOI:** 10.1371/journal.pone.0337757

**Published:** 2025-12-01

**Authors:** Xin Xu, Ghada Homsi, Sherry T. Liu, Jennifer M. Gaber, Naa A. Inyang, Brian L. Rostron, Caryn F. Nagler, James Nonnemaker

**Affiliations:** 1 Center for Tobacco Products, U.S. Food and Drug Administration, Silver Spring, Maryland, United States of America; 2 RTI International, Research Triangle Park, North Carolina, United States of America; Hospital Infantil de México Federico Gomez: Hospital Infantil de Mexico Federico Gomez, MEXICO

## Abstract

**Background:**

In 2022, 3.7% of U.S. adults currently smoked cigars. This study assesses cigar-smoking-attributable fractions in U.S. healthcare expenditures and associated annual healthcare expenditures overall and by payer, including publicly funded healthcare programs.

**Methods:**

Data were obtained from the 2000, 2005, 2010, and 2015–2017 National Health Interview Survey linked with corresponding panels from the Medical Expenditure Panel Survey data through 2018. The final sample (n = 53,733) was restricted to adults aged 25 + . Estimates from four-part models and data from the Personal Health Care component of the 2001–2018 National Health Expenditures Accounts were combined to estimate fractions of and annual healthcare expenditures attributable to cigar smoking. All models controlled for sociodemographic characteristics and health-related behaviors.

**Results:**

During 2001–2018, an estimated 1.8% (95% CI = 0.9%–3.4%) or $29.7 billion annually of U.S. healthcare expenditures could be attributed to cigar smoking. Most of this was funded by other third-party health insurance programs, a mix of private and public payers (e.g., Department of Veterans Affairs).

**Conclusions:**

Cigar smoking creates a preventable financial burden on the U.S. healthcare system. Health consequences associated with cigar smoking may remain after successful quitting. The findings underscore the importance of preventing initiation of cigar smoking and providing evidence-based cessation methods to reduce the health and economic burden of cigar smoking.

## Introduction

In 2022, 10.2 million U.S. adults aged 18 years and older reported cigar use in the past month, and 1.3 million had initiated use in the past year [1]. An estimated 3.7% of U.S. adults currently smoke cigars every day or some days [2]. Each year, regular cigar use is estimated to cause 9,000 premature deaths—a loss of 140,000 life-years [3]—among U.S. adults aged 35 years and older, as cigar smoking has many of the same health risks as cigarette smoking, including coronary heart disease, aortic aneurysm, and cancers of the oral cavity, esophagus, pancreas, larynx, and lung [4–6]. Compared to cigarettes, the variety of cigar flavors and smaller, cheaper packs have been found to increase appeal and sales among consumers, including youth and young adults [7–9]. Although current cigar use among youth and young adults has decreased over time, it has remained steady among adults 25 years of age and older [10,11]. Uptake and use of these combustible products continue to put U.S. public health at risk [10,12].

It is estimated that preventable risk factors (among which the use of combustible tobacco products are the top contributor) are associated with more than one-fourth of all U.S. healthcare expenditures [13]. Although numerous studies have assessed cigarette-smoking-attributable healthcare expenditures [14–20], few have quantified such healthcare expenditures attributable specifically to cigar smoking. Using average unit costs of certain medical visits, Wang and colleagues [21] found that annual healthcare expenditures attributable to exclusive cigar smoking in the U.S. were $284 million between 2000 and 2015 [21]. Total annual healthcare expenditures attributable to cigar smoking were an estimated $1.8 billion [21].

However, no studies to date have assessed differences in U.S. healthcare expenditures attributable to cigar smoking by payer. In the U.S., personal healthcare utilizations are reimbursed by a variety of payer sources, including private insurance (e.g., employer-based health insurance), public insurance programs (e.g., Medicaid and Medicare), out-of-pocket (e.g., paid directly by the user), and other third-party payers (including the Department of Veterans Affairs/Civilian Health and Medical Program of the Department of Veterans Affairs (CHAMPVA); TRICARE [a U.S. healthcare program for active duty and retired military and their families]; other federal sources; other state and local sources; Workers’ Compensation; other public sources; and other unclassified sources). Medicaid, codified under Title XIX of the Social Security Act and managed by state governments with both federal and state funds, is a government health insurance program that provides approved medical assistance to individuals with limited income and resources. Medicare, enacted under Title XVIII of the Social Security Act, is a federal health insurance program to provide approved medical services to individuals aged 65 or older and younger people with disabilities. Public health insurance programs of Medicaid and Medicare covered an estimated 40.3% of total personal healthcare expenditures in 2019, while private health insurance was the largest single payer, accounting for 33.2% of the total, followed by 13.8% from other third-party payers and 12.7% from out-of-pocket [22]. Given the complexity of the U.S. healthcare payment system, understanding the payers of cigar-attributable expenditures is critical. It provides both private insurers and government payers with economic justification for investing in cessation interventions and prevention programs [23,24].

Recent studies have shown that an estimated 12%–15% of U.S. annual healthcare expenditures could be attributed to cigarette smoking [14–19]. Xu and colleagues [18] found that 50.0% of these cigarette-attributable healthcare expenditures were funded by public payers, such as Medicare or Medicaid. However, it is unknown to what extent each healthcare payer group is associated with cigar-smoking-attributable expenditures and whether the burden falls mainly on public or private payers.

Given the persistent prevalence of cigar use [1,25,26] and the literature gap, the objective of this study is to provide more recent estimates of cigar-smoking-attributable healthcare expenditures overall, as well as the first known estimates by payer group (Medicare, Medicaid, private insurance, out-of-pocket, and other third-party payers). Findings can provide information on the healthcare expenditures associated with cigar use during 2001–2018 and offer insights into the differential burden of expenditures covered by public health insurance programs compared to those covered by private health insurance. This study underscores the potential savings from reduced healthcare utilization, overall and by payer, that could result from cigar use prevention and cessation efforts.

## Materials and methods

This study utilized an established econometric approach to estimate annual direct healthcare expenditures and attributable fractions associated with cigar smoking, both overall and by payer [27].

The fundamental principle underlying this approach involves creating an appropriate counterfactual—a comparison group of individuals who never smoked cigars but possess similar characteristics to typical cigar smokers [27]. This methodological consideration is critical because not all observable differences in healthcare expenditures between smokers and non-smokers are attributable to cigar smoking, particularly given the potential for dual use of cigarettes and cigars. To isolate expenditures appropriately attributable to cigar smoking, the regression model incorporated comprehensive sociodemographic controls and other risky behaviors, including cigarette use variables, to account for observable differences between cigar smokers and non-cigar smokers.

This approach offers a key advantage by comparing the costs of ever, current, and former users to never users without requiring an exhaustive list of diseases related to cigar smoking. Rather than aggregating direct healthcare costs for specific cigar-related diseases, this method estimates the direct healthcare cost of cigar smoking by calculating the difference in healthcare costs between cigar smokers and never users, adjusted for confounders. By estimating the annual direct healthcare costs associated with cigar smoking, this approach is well-suited for understanding how these costs are distributed across healthcare payers.

### Data sources

Data from the 2001, 2002, 2006, 2007, 2011, 2012, and 2016–2018 Medical Expenditure Panel Survey (MEPS) Household Component were linked with the 2000, 2005, 2010, and 2015–2017 National Health Interview Survey (NHIS).

MEPS is an in-person household interview survey that provides nationally representative estimates for healthcare use, expenditures, sources of payment, and health insurance coverage for the U.S. civilian non-institutionalized population. This excludes those living in long-term care facilities, correctional facilities, and those who are active-duty military personnel [28]. Expenditures data in MEPS are based on direct payments provided for respondents’ healthcare services during the prior 12 months. Each MEPS full-year consolidated data file is drawn from the households that participated in NHIS during the two preceding years.

NHIS is an annual cross-sectional, in-person household interview survey of the U.S. civilian non-institutionalized population on health-related topics. Prior to 2016, cigar smoking data were collected every 5 years in the Cancer Control Supplement to the NHIS, which included questions on cancer-related knowledge, attitudes, and health behaviors. Beginning in 2016, cigar smoking questions were included in the annual NHIS survey. MEPS data were linked to NHIS data using confidential MEPS-NHIS linkage files maintained by the Agency for Healthcare Research and Quality (AHRQ) Data Center and accessed through a restricted data application process. (a conceptual diagram of data linkage and study design is provided in S1 Appendix).

Estimated healthcare expenditures reported in the MEPS data can be lower than personal healthcare expenditures reported by the Centers for Medicare & Medicaid Services [29–32]. To avoid underestimates, this study used annual U.S. healthcare expenditures from the Personal Health Care (PHC) component of the 2001–2018 National Health Expenditures Accounts (NHEA) data to calculate the average annual healthcare expenditures attributable to cigar smoking in the U.S. by incorporating our estimates of attributable fractions from the econometric approach described in the section of the four-part model below [33]. The PHC is considered the official estimate of total healthcare expenditures in the U.S.; the PHC Price Index was used to adjust all monetary variables to real 2018 U.S. dollars [34].

The Institutional Review Board of RTI International determined that this study was not research involving human subjects, and all data were fully anonymized.

#### Sample selection.

Because the NHIS question about lifetime use of 50 or more cigars was not included after 2015 and recent studies have shown that cigar smoking initiation typically starts at later age, the pooled NHIS-MEPS sample was restricted to respondents aged 25 and older at the time of the NHIS interview to better capture established cigar smokers instead of experimenters [35,36]. The final sample size was 53,733 respondents after excluding those with missing cigar-smoking status (1,691). Among them, an additional 942 respondents with missing data on demographic and health behavior variables were removed when those variables were used in the analysis.

#### Variable definitions.

Respondents were categorized into three cigar-smoking status groups based on self-reported use of large cigars, cigarillos, or little filtered cigars in NHIS: (1) current cigar smokers who smoked cigars some days or every day; (2) former cigar smokers who ever smoked cigars in their lifetime but were not smoking cigars at all currently; and (3) lifetime never cigar smokers. Those who were either current or former cigar smokers were considered ever cigar smokers.

Respondents were classified into three cigarette-smoking status groups: (1) current cigarette smokers who had smoked 100 cigarettes in their lifetime and smoked cigarettes some days or every day at the time of interview; (2) former cigarette smokers who had smoked 100 cigarettes in their lifetime but were not smoking cigarettes at all at the time of interview; and (3) never cigarette smokers who had not smoked 100 cigarettes in their lifetime.

Both alcohol consumption status (current drinker, former drinker, lifetime abstainer) and past-12-month receipt of an influenza vaccine (yes, no) were included as proxy variables for other risky behaviors.

Demographic variables included sex (male, female); age group (25–44, 45–64, 65–74, 75+); race/ethnicity (non-Hispanic white, non-Hispanic Black, Hispanic, non-Hispanic other); education (less than high school diploma, high school diploma or GED, some college or an associate degree, bachelor’s degree or above); and marital status (married, never married, divorced/separated/widowed) (see Table A in S3 Appendix for more detailed definitions and data sources of these variables).

The dependent variable in each model was on annual individual healthcare expenditures from MEPS. MEPS individual-level healthcare expenditure information and health insurance coverage were used to define five payer groups: Medicare, Medicaid, private insurance, out-of-pocket, and other third-party payers (Table B in S3 Appendix). Because respondents could have multiple types of payment sources within a given year, we also constructed three mutually exclusive groups based on the presence of healthcare payments and the number of payer groups involved (henceforth, “payer mix”): respondents with (1) no reported healthcare expenditures, (2) positive healthcare expenditures paid by one payer, and (3) positive healthcare expenditures paid by multiple payer groups (Table C in S3 Appendix).

### Statistical analysis

#### Sample weighting and descriptive analyses.

To account for non-linkable NHIS-MEPS respondents and present nationally representative estimates of the noninstitutionalized U.S. adult population, the individual-level analytic weights of the study sample were adjusted (S2 Appendix). Weighted descriptive statistics were conducted to provide nationally representative estimates of adults’ cigar use, cigarette use, demographics, and health-related behaviors, overall and by each payer group. Similar descriptive statistics were conducted by payer mix.

#### Econometric model selection: Two- vs. four-part models.

To assess the attributable fraction of healthcare expenditures associated with cigar smoking, we explored two different models (two- vs. four-part models) for the econometric approach to estimate healthcare expenditures as a function of cigar-smoking status [18,37], while controlling for comprehensive sociodemographic variables and other risky behaviors. [15,27]. Copas tests were used to evaluate over-fitting and misspecification among the two- and four-part models, and the four-part model was determined to be a better fit (S1 Appendix) [19,38]. Therefore, four-part model results are presented here.

#### Four-part model.

Empirically, a four-part model was used in the cross-sectional regression approach. In a four-part model, logistic regressions were conducted in the first and third parts to estimate the probability of having positive annual healthcare expenditures and positive inpatient expenditures, respectively. In the second and fourth parts of the model, generalized linear regressions with a log link and gamma distribution were conducted to assess the amount of healthcare expenditures, conditioned on having positive expenditures and no inpatient expenditures, and then on having positive inpatient expenditures, respectively. Using predicted values from each of the four parts of the model, the difference between predicted healthcare expenditures for cigar smokers and their predicted expenditures had they never smoked cigars was calculated. The cigar-smoking-attributable expenditures were divided by total predicted healthcare expenditures for the entire population to estimate cigar-smoking-attributable fractions, overall and by payer (see S1 Appendix for equations and additional details). Total healthcare expenditures attributable to cigar smoking were estimated by multiplying the cigar-smoking-attributable fractions calculated from the MEPS data by the annual NHEA–PHC healthcare expenditure data, overall and by payer.

The bootstrap method was used to calculate standard errors and 95% confidence intervals (CIs). Estimated attributable fractions and expenditures were considered statistically significant if the bootstrapped 95% CIs did not include zero. SAS version 9.4 was used to adjust the analytic weight and Stata version 15.1 was used to conduct weighted analyses during 2020–2024.

### Sensitivity analysis

#### Primary payer group.

To assess the robustness of our findings, we conducted two types of sensitivity analyses. The first sensitivity analysis was conducted to assess whether results by payer group were sensitive to overlap of individuals across payers. In the main analysis, payers were defined based on respondents’ actual payer of each healthcare service within each MEPS year. Therefore, each respondent could belong to multiple payer groups during a year if more than one payer paid for the respondent’s medical services. To ensure mutual exclusivity of individuals across payer groups in this sensitivity analysis, the primary payer of each respondent was defined using a “majority rule”; that is, the primary payer was defined as the payer group that paid for more than half of a person’s total past-12-month expenditures (Table B in S3 Appendix).

#### Lifetime threshold of cigar use.

A second sensitivity analysis was conducted to assess the impact of incorporating a lifetime cigar use threshold to define current and former users. The NHIS question about lifetime use of 50 or more cigars was not asked after 2015. Therefore, the lifetime threshold was not incorporated into the cigar-smoking status definitions used in the main analysis. In this sensitivity analysis, we used a sub-sample from NHIS-linked MEPS data between 2001 and 2016 when the lifetime threshold of cigar use variable was available. We further defined a four-category cigar smoking status variable where former cigar smokers were split into former experimenters (used < 50 cigars in lifetime) or former regular users (used 50 + cigars in lifetime). We assessed differences in weighted prevalence and cigar-attributable fractions between former regular cigar smokers and former cigar experimenters. Given the small sample size in NHIS-linked MEPS data from 2001–2016, we did not conduct this sensitivity analysis separately by payer.

## Results

### Descriptive analysis results

Overall, 29.1% of U.S. adults aged 25 and older were ever cigar smokers, of which 3.8% were current cigar smokers, and 25.3% were former cigar smokers (Table 1). By payer, the ever cigar-smoking prevalence was highest among those with any healthcare expenditures from other third-party payers (32.7%), followed by private insurance (29.5%), out-of-pocket (29.1%), Medicare (25.8%), and Medicaid (23.8%). The ever cigar-smoking prevalence was more comparable across healthcare payer mix categories: 30.7% among those with no reported expenditures, 29.9% among those with a single payer, and 28.9% among those with multiple payers (Table 2). Approximately 90.0% of MEPS respondents had more than one payer within a calendar year (Table C in S3 Appendix). Annual estimates of weighted cigar smoking prevalence ranged from 3.4% to 4.6% (Table D in S3 Appendix). Pooled estimates of cigar and cigarette smoking prevalence overall and by payer are in Table 1, and by payer mix in Table 2; descriptive statistics of individuals’ sociodemographic characteristics are in Table E (by payer) and Table F (by payer mix) in S3 Appendix.

**Table 1:** Cigar and cigarette smoking prevalence, overall and by payer, NHIS-linked MEPS 2001–2018.

Smoking Status	Overall	Medicare^a^	Medicaid^a^	Private^a^	Out-of-Pocket^a^	Other Third-Party^a, b^
	n = 53,733	n = 16,725	n = 8,915	n = 37,065	n = 51,298	n = 13,564
	Weighted % (95% CI)^c^					
Ever cigar-smoking status						
Ever	29.1 (28.7, 29.6)	25.8 (24.9, 26.6)	23.8 (22.7, 25.0)	29.5 (29, 30.1)	29.1 (28.6, 29.6)	32.7 (31.7, 33.7)
Never	70.9 (70.4, 71.3)	74.2 (73.4, 75.1)	76.2 (75.0, 77.3)	70.5 (69.9, 71.0)	70.9 (70.4, 71.4)	67.3 (66.3, 68.3)
Current cigar-smoking status						
Current	3.8 (3.6, 4.1)	2.2 (1.9, 2.5)	3.6 (3.1, 4.1)	3.7 (3.4, 3.9)	3.8 (3.6, 4.0)	4.4 (4.0, 4.9)
Former	25.3 (24.8, 25.8)	23.5 (22.7, 24.4)	20.2 (19.2, 21.3)	25.9 (25.3, 26.4)	25.3 (24.8, 25.7)	28.2 (27.3, 29.2)
Never	70.9 (70.4, 71.3)	74.2 (73.4, 75.1)	76.2 (75.0, 77.3)	70.5 (69.9, 71.0)	70.9 (70.4, 71.4)	67.3 (66.3, 68.3)
Ever cigarette-smoking status						
Ever	43.0 (42.5, 43.6)	50.5 (49.6, 51.4)	50.4 (49.1, 51.8)	40.8 (40.2, 41.4)	43.3 (42.7, 43.8)	48.4 (47.3, 49.4)
Never	57.0 (56.4, 57.5)	49.5 (48.6, 50.4)	49.6 (48.2, 50.9)	59.2 (58.6, 59.8)	56.7 (56.2, 57.3)	51.6 (50.6, 52.7)
Current cigarette-smoking status						
Current	19.2 (18.8, 19.6)	13.0 (12.4, 13.6)	29.8 (28.6, 31.0)	16.6 (16.1, 17.0)	19.2 (18.8, 19.6)	22.0 (21.1, 22.8)
Former	23.8 (23.4, 24.3)	37.5 (36.6, 38.4)	20.6 (19.6, 21.7)	24.3 (23.7, 24.8)	24.1 (23.6, 24.5)	26.4 (25.5, 27.3)
Never	57.0 (56.4, 57.5)	49.5 (48.6, 50.4)	49.6 (48.2, 50.9)	59.2 (58.6, 59.8)	56.7 (56.2, 57.3)	51.6 (50.6, 52.7)

Notes: Weighted estimates are for U.S. adults aged 25 years and older. Smoking status was defined based on National Health Interview Survey questions, which vary across survey years (https://www.cdc.gov/nchs/nhis/tobacco/tobacco_questions.htm).

^a^The respondents within each payer group are not mutually exclusive, because respondents can have multiple payers within a calendar year.

^b^The Medical Expenditure Panel Survey definition for “other third-party” includes Veterans Administration/CHAMPVA, TRICARE, other federal sources, other state and local sources, Workers’ Compensation, other public, and other unclassified sources.

^c^The proportions of the categories of each variable are based on the number of respondents with non-missing values for that variable in the sample.

**Table 2:** Cigar and cigarette smoking prevalence, overall and by healthcare payer mix.

Smoking Status	Overall	No Reported Healthcare Expenditure^a, b^	Single Payer (Any)^b, c^	Multiple Payers^b, d^
	n = 53,733	n = 6,315	n = 5,076	n = 42,342
	Weighted % (95% CI)^e^			
Ever cigar-smoking status				
Ever	29.1 (28.7, 29.6)	30.7 (29.2, 32.2)	29.9 (28.3, 31.5)	28.9 (28.3, 29.4)
Never	70.9 (70.4, 71.3)	69.3 (67.8, 70.8)	70.1 (68.5, 71.8)	71.1 (70.6, 71.7)
Current cigar-smoking status				
Current	3.8 (3.6, 4.1)	5.2 (4.5, 5.9)	5.2 (4.4, 6.1)	3.5 (3.3, 3.8)
Former	25.3 (24.8, 25.8)	25.5 (24.1, 26.9)	24.7 (23.1, 26.3)	25.4 (24.8, 25.9)
Never	70.9 (70.4, 71.3)	69.3 (67.8, 70.8)	70.1 (68.4, 71.7)	71.1 (70.6, 71.7)
Ever cigarette-smoking status				
Ever	43.0 (42.5, 43.6)	44.3 (42.7, 45.9)	42.0 (40.3, 43.8)	43.0 (42.4, 43.6)
Never	57.0 (56.4, 57.5)	55.7 (54.1, 57.3)	58.0 (56.2, 59.7)	57.0 (56.4, 57.6)
Current cigarette-smoking status				
Current	19.2 (18.8, 19.6)	28.4 (27 29.8)	25.7 (24.2, 27.3)	17.3 (16.8, 17.7)
Former	23.8 (23.4, 24.3)	15.9 (14.8, 17.1)	16.3 (15.1, 17.6)	25.7 (25.2, 26.2)
Never	57.0 (56.4, 57.5)	55.7 (54.1, 57.3)	58.0 (56.3, 59.7)	57.0 (56.4, 57.6)

Notes: Weighted estimates are for U.S. adults aged 25 years and older, National Health Interview Survey-linked Medical Expenditure Panel Survey 2001–2018.

^a^Individuals who did not report using any medical services, including annual physical exams, in the past year; therefore, they did not incur any medical expenditures in the past year.

^b^Respondents within each healthcare payer mix category are mutually exclusive.

^c^“Single payer” is defined as having healthcare expenditures from only one of the five primary payer groups: Medicare, Medicaid, private insurance, out-of-pocket, or other third-party within a calendar year.

^d^“Multiple payers” is defined as having healthcare expenditures from at least two of the five primary payer groups in a calendar year.

^e^The proportions of the categories of each variable are based on the number of respondents with non-missing values for that variable in the sample.

### Four-part model results

Based on the four-part model in the main analysis, an estimated 1.8% (95% CI = 0.9%–3.4%) of U.S. direct healthcare expenditures ($29.7 billion annually) could be attributed to cigar smoking. Most of this was associated with former cigar smoking (1.6%; 95% CI = 0.1%–3.1%, or $26.4 billion annually) (Table 3). By payer, 12.7% (95% CI = 6.9%–18.5%, $29 billion annually) of healthcare expenditures paid by other third-party payers could be attributed to ever cigar smoking. Cigar-smoking-attributable fractions for Medicare, Medicaid, private insurance, and out-of-pocket were not statistically significant, as shown in Table 3. Therefore, we cannot reject the null hypotheses (that cigar smoking was not associated with healthcare expenditures by these payers) and we do not report annual cigar-smoking-attributable healthcare expenditures for these payers. Two-part model results are presented in Table H in S3 Appendix for comparison.

**Table 3:** Cigar-smoking-attributable fractions estimated from the four-part model and annual healthcare expenditures, overall and by payer.

Cigar-Smoking Status by Payer	Weighted % (95% CI)	
	Percent Attributable Fraction	NHEA 2001–2018 ($ billions)^a^
**Total expenditure (n = 52,791)** ^**b**^		
Ever	**1.8 (0.9–3.4)**	29.7 (5.4–54.1)
Current	0.2 (−0.1–0.5)	—
Former	**1.6 (0.1–3.1)**	26.4 (3.2–49.7)
**Medicare (n = 16,375)**		
Ever	−1.5 (−3.8–0.9)	—
Current	−0.1 (−0.5–0.4)	—
Former	−1.4 (−3.6–0.8)	—
**Medicaid (n = 8,681)**		
Ever	1.0 (−2.5–4.4)	—
Current	−0.2 (−1.1–0.7)	—
Former	1.2 (−2.1–4.5)	—
**Private (n = 36,549)**		
Ever	1.9 (−0.1–3.9)	—
Current	0.2 (−0.4–0.7)	—
Former	1.7 (−0.2–3.6)	—
**Out-of-pocket (n = 50,411)**		
Ever	0.7 (−0.8–2.3)	—
Current	0.1 (−0.3–0.4)	—
Former	0.7 (−0.6–2.0)	—
**Other third-party (n = 13,299)** ^**c**^		
Ever	**12.7 (6.9–18.5)**	28.9 (15.7–42.3)
Current	**1.9 (0.1–3.7)**	4.4 (3.4–8.5)
Former	**10.8 (5.3–16.2)**	24.6 (12.2–37.0)

Notes: Weighted estimates are for U.S. adults aged 25 years and older, National Health Interview Survey-linked Medical Expenditure Panel Survey 2001–2018. Boldface indicates statistical significance (p < 0.05) based on bootstrapped 95% CIs.

^a^Dollar values were adjusted to 2018 dollars using the Personal Health Care Price Index.

^b^Sample size excluded 942 individuals who had missing values for the regression covariates.

^c^The National Health Expenditure Accounts definition for “other payers” includes other health insurance programs (Children’s Health Insurance Program [Titles XIX and XXI], Department of Defense, and Department of Veterans Affairs) and other third-party payers (worksite healthcare, other private revenues, Indian Health Service, workers’ compensation, general assistance, maternal and child health, vocational rehabilitation, other federal programs, Substance Abuse and Mental Health Services Administration, other state and local programs, and school health).

### Sensitivity analysis: Primary payer results

For the first sensitivity analysis based on the primary payer approach, all weighted descriptive statistics by primary payer group are reported in Table G in S3 Appendix. Our findings from the four-part model among each primary payer group suggest that 2.5% (95% CI = 0.2%–4.9%, or $14.1 billion) of healthcare expenditures among individuals with most of their expenditures paid by private insurance, and 8.5% (95% CI = 0.8%–16.2%, or $19.4 billion) of expenditures for individuals with most of their payment covered by other third-party payers, could be attributed to cigar smoking. Cigar-smoking-attributable fractions for primary payer groups of Medicare, Medicaid, and out-of-pocket remained insignificant, so annual cigar-smoking-attributable expenditures are not reported for these (Table 4).

**Table 4:** Primary payer sensitivity analysis: cigar-smoking-attributable fractions estimated from the four-part model and annual healthcare expenditures, overall and by primary payer.

Cigar-Smoking Status by Primary Payer	Weighted % (95% CI)	
	Percent Attributable Fraction	NHEA 2001–2018 ($ billions)^a^
**Total expenditure (n = 52,791)** ^**b**^		
Ever	**1.8 (0.9–3.4)**	29.7 (5.4–54.1)
Current	0.2 (−0.1–0.5)	—
Former	**1.6 (0.1–3.1)**	26.4 (3.2–49.7)
**Medicare (n = 9,735)**		
Ever	−2.0 (−4.3–0.2)	—
Current	−0.4 (−0.8–0.0)	—
Former	−1.7 (−3.8–0.4)	—
**Medicaid (n = 4,815)**		
Ever	1.5 (−2.4–5.4)	—
Current	−0.1 (−1.1–0.9)	—
Former	1.6 (−2.0–5.2)	—
**Private (n = 20,811)**		
Ever	**2.5 (0.2–4.9)**	14.1 (1.0–27.2)
Current	0.2 (−0.3–0.7)	—
Former	**2.3 (0.2–4.5)**	13.1 (1.0–25.1)
**Out-of-pocket (n = 16,060)**		
Ever	0.9 (−0.0–3.6)	—
Current	0.2 (−0.4–0.8)	—
Former	0.7 (−1.9–3.3)	—
**Other third-party (n = 6,580)** ^**c**^		
Ever	**8.5 (0.8–16.2)**	19.4 (1.8–37.1)
Current	2.4 (−0.1–4.8)	—
Former	6.0 (−0.8–12.7)	—

Notes: Weighted estimates are for U.S. adults aged 25 years and older, National Health Interview Survey-linked Medical Expenditure Panel Survey 2001–2018. Boldface indicates statistical significance (*p* < 0.05) based on bootstrapped 95% CIs.

^a^Dollar values were adjusted to 2018 dollars using the Personal Health Care Price Index.

^b^Sample size excluded 942 individuals who had missing values for the regression covariates.

^c^The National Health Expenditure Accounts (NHEA) definition for “other payers” includes other health insurance programs (CHIP [Titles XIX and XXI], Department of Defense, and Department of Veterans Affairs) and other third-party payers (worksite healthcare, other private revenues, Indian Health Service, workers’ compensation, general assistance, maternal and child health, vocational rehabilitation, other federal programs, Substance Abuse and Mental Health Services Administration, other state and local programs, and school health).

### Sensitivity analysis: Lifetime threshold of cigar use results

In the second sensitivity analysis, which used a lifetime cigar use threshold for defining an alternate four-category cigar smoking variable, and a subsample from NHIS-linked MEPS data between 2001 and 2016 (N = 34,959), 28.7% of respondents were ever cigar smokers, including 19.9% former cigar experimenters, 4.9% former regular cigar smokers, and 3.9% current cigar smokers (results not shown). The four-part model results from the cigar threshold sensitivity analysis showed that 2.4% (95% CI = 0.8%–4.0%) of total healthcare expenditures ($37.5 billion annually) could be attributed to cigar smoking, with an estimated 1.3% (95% CI = 0.1%–2.6%) from former cigar experimenters and 0.8% (95% CI = 0.1%–1.6%) from former regular cigar smokers (Table I in S3 Appendix).

## Discussion

Cigar smoking accounted for approximately 1.8% of total direct healthcare expenditures in the U.S. from 2001 to 2018, which represents an estimated annual expenditure of $29.7 billion based on average annual personal healthcare expenditures reported during this time. For every $100 spent on healthcare each year among adults aged 25 and older, almost $1.80 can be attributed to cigar smoking.

Unlike healthcare expenditures attributable to cigarette smoking in the U.S., where more than 50% of attributable expenditures was paid through Medicaid and Medicare [18,19], we did not find evidence suggesting that Medicaid and Medicare healthcare expenditures were significantly associated with cigar smoking. Instead, the main analysis suggests that more than 95% of the cigar-smoking-attributable healthcare expenditures were paid by other third-party health insurance: a mix of public and private payers that includes the Department of Veterans Affairs. According to data from the 2010–2015 National Survey on Drug Use and Health, 6.2% of the U.S. military veteran population smoked cigars [39], which is two and a half percentage points higher than the national rate [2]. This high cigar smoking prevalence among veterans may contribute to the disproportionate burden of cigar-smoking-attributable healthcare expenditures among the other third-party payer group.

In the primary payer sensitivity analysis, we found that approximately 60% of expenditures were incurred by respondents whose primary payer group was other third-party payers, and the remaining 40% came from those whose primary payer was private insurance. These findings are unsurprising, since the ever cigar-smoking prevalence among respondents with other third-party insurance as the primary payer was approximately 45% higher than those with Medicaid, and approximately 37% higher than those with Medicare. Compared to those with Medicaid and Medicare as the primary payers, the ever cigar-smoking prevalence among respondents with private insurance as the primary payer were approximately 29% and 22% higher, respectively. In contrast, cigarette-smoking prevalence was lower among those with other third-party insurance programs and private insurance compared to those with Medicaid/Medicare as the primary payers; the ever cigarette-smoking prevalence was approximately 9% or 8% lower among those with other third-party insurance programs and approximately 26% or 24% lower among those with private insurance as the primary payer (Table G in S3 Appendix). Therefore, variations in cigar- and cigarette-smoking prevalence across insurance programs likely contributed to different attributable fraction findings by payer for cigarettes and cigars.

The different findings between the main analysis and primary payer sensitivity analysis are likely due to respondents with multiple insurance program coverage, particularly those who had both private insurance and other third-party insurance expenditures within the same year (11.8% of respondents; results not shown). Following the existing literature [16,19,25] the main analysis stratified healthcare expenditures reimbursed by payers to better articulate the cigar smoking–associated expenditures for each payer. However, one limitation of this approach is that an individual with multiple healthcare payment methods (e.g., has both Medicare and private insurance coverages) would be included in multiple regressions depending on payers used for these medical services, and none of the regressions by payer captures the overall annual healthcare expenditures of this individual. To address this issue, the majority rule was used in the sensitivity analysis to create mutual exclusivity at the individual level.

Another unique feature is that former cigar smoking accounted for more than 80% of the burden, while current cigar smoking was not significantly associated with healthcare expenditures. This finding, which remains consistent in the primary and sensitivity analyses and across different payers and study years, seems reasonable, as the proportion of former cigar smokers in the final sample was roughly six times that of current cigar smokers (25.3% vs. 3.8%, Table 1). Additionally, most (69%) ever cigar smokers were former experimenters. However, former smokers who used cigars regularly accounted for 34% of total cigar-attributable expenditures, despite making up just 17% of the population who had ever smoked cigars, suggesting that the per-person healthcare expenditures associated with former regular cigar smokers may be much larger than that associated with former cigar experimenters. This is consistent with the findings from existing literature on cigar-smoking-related mortality, indicating that former cigar smoking was significantly associated with higher prevalence of smoking attributable diseases, including heart conditions, stroke, and cancer [33]. This finding is also likely associated with sustained impacts of cigar smoking on individual health even after successfully quitting, as well as the “sick quitter effect,” where a recent successful quit is caused by the onset of adverse symptoms or newly diagnosed disease [40–44]. Additional research fully examining the underlying drivers of these observed patterns would be informative.

Although findings from this analysis and the other existing study suggest that cigar smoking is associated with substantial healthcare utilization and expenditures, the estimated attributable annual healthcare expenditure differs [21]. This is likely due to different methods used, although both studies used the same data sources and identified relative risks associated with “all-causes” cigar use. The key difference between these two methods is estimating relative risks of cigar-smoking-attributable healthcare utilization [21] versus relative risks (namely attributable fractions) of healthcare expenditures used in this study. Wang and colleagues first estimated relative risks of cigar-smoking-attributable healthcare utilization, including hospital nights, emergency department (ED) visits, physician visits, and home-care visits, from the 2000, 2005, 2010, and 2015 NHIS data, then applied these relative risks of healthcare utilization to average healthcare expenditures of these utilizations estimated from MEPS. The benefit of this method is that it does not require data linkage between NHIS and MEPS. In addition, their approach allows for estimates by exclusive and poly cigar smokers.

In contrast, our method directly estimated the attributable fractions for cigar-smoking-related healthcare expenditures, which requires the data linkage of NHIS and MEPS. This approach is typically used by existing studies to estimate cigarette-smoking attributable healthcare expenditures [13–20,27], as it offers several other advantages. First, instead of using average unit costs of hospital stays, ED visits, physician visits, and homecare visits across MEPS respondents, this analysis is based on individual annual healthcare expenditures, which includes all medical utilization in the past 12 months. Therefore, it may cover additional healthcare utilization and expenditures, such as prescription drugs or outpatient exams, which are not captured in Wang and colleagues’ estimates. Second, this approach relaxes the assumption in Wang and colleagues that the cigar-smoking-attributable fraction of medical utilization would be the same as that of medical expenditures, because medical expenditures could vary widely for the same medical procedure, depending on individual comorbidities. This study directly estimated the attributable fractions for cigar-smoking-related healthcare expenditures to avoid this issue. In addition, the attributable expenditures estimated in this analysis were based on the PHC expenditures reported in NHEA. These were used to address potential underestimations of healthcare expenditures in MEPS, as studies have shown that MEPS data do not include healthcare expenditures for institutionalized populations, long-term care greater than 445 days, active-duty military personnel, or certain types of healthcare (e.g., over-the-counter medications) [29–32] and thus can underestimate actual healthcare expenditures. Also, unlike Wang’s study, this analysis includes a young adult population aged 25–34 years, given that the short-term health risks—particularly cardiovascular—of cigar smoking are well documented in the literature [45–47]. Finally, this analysis is based on a more recent NHIS and MEPS sample.

### Limitations

This analysis is subject to a few limitations. The estimated cigar-smoking-attributable healthcare expenditures are likely to be conservative because this analysis does not consider additional healthcare expenditures associated with secondhand cigar smoke or prenatal care [48–50], nor does it estimate indirect expenditures or societal costs associated with cigar smoking. The findings may be subject to recall bias from either cigar-smoking history in NHIS or self-reported healthcare use in MEPS. NHIS did not ask respondents about cigar type ever smoked and paused the question on number of cigars smoked in lifetime after 2015. Therefore, both current and former cigar smokers in the main analysis can be heterogeneous, as lifetime cigar use could not be used to distinguish regular smokers from experimenters or to identify the type of cigar smoked. However, our sensitivity analysis based on the NHIS-linked MEPS data between 2001 and 2016 suggests that former cigar experimenters—those who self-reported having used less than 50 cigars in their lifetime—also experienced health consequences associated with cigar smoking, although the average per-person healthcare expenditure was much higher among regular cigar smokers. These findings are consistent with findings from cigarette smoking that long-term health risks exist for even light and intermittent tobacco use [51,52]. Additionally, the exclusion of adults 18–24 years of age from this analysis may have resulted in a slight underestimation of healthcare expenditures, although young adult healthcare expenditures are expected to be low. NHIS also did not consistently ask respondents about tobacco product use other than cigarettes and cigars, so the use of other products could not be identified. Additionally, self-reported cigar-smoking status comes from NHIS, while healthcare expenditure data come from MEPS. Therefore, the time lag associated with the MEPS sampling frame may introduce a misclassification of current cigar-smoking status, as it is possible that current cigar smokers at the time of the NHIS interview might have quit before taking the follow-up MEPS survey. This is unlikely to bias results if recent quitters and continued cigar users have similar medical expenditures. Recent quitters and continuing cigarette smokers were found to have similar expenditures in a cigarette-smoking cessation study [42]. Finally, by applying the cigar-smoking-attributable fractions estimated from MEPS to the annual PHC from NHEA, there is an assumption that the active-duty and institutionalized U.S. populations share the same attributable fractions of cigar smoking as the civilian non-institutionalized U.S. population.

## Conclusions

This study provides a more recent and more comprehensive estimate of healthcare expenditures attributable to cigar smoking among U.S. adults between 2001 and 2018, overall and by payer. It clearly shows that, with annual expenditures of almost $30 billion, cigar smoking creates a preventable financial burden on the U.S. healthcare system. This financial burden was disproportionately carried by other third-party health insurance programs, which include a mix of public and private payers such as the Department of Veterans Affairs. In addition, this study suggests that sustained health consequences associated with cigar smoking may remain after successful quitting. This underscores the importance of preventing cigar smoking initiation and providing evidence-based methods to support cigar smoking cessation as early in life as possible to reduce the health and economic burden of cigar smoking in the U.S.

## Supporting information

S1 Appendix Calculating cigar-smoking-attributable fractions in healthcare expenditures and annual healthcare expenditure estimates. (DOCX)

S2 Appendix NHIS-MEPS individual-level weight adjustment. (DOCX)

S3 Appendix Additional results. (DOCX)
