# Supplementary material for: Annual U.S. healthcare expenditures attributable to cigar smoking between 2001 and 2018, overall and by payer
Source: PLoS One. 2025 Dec 1;20(12):e0337757. doi: 10.1371/journal.pone.0337757 (PMC12668525; doi:10.1371/journal.pone.0337757)
Supplement: S1 Appendix — (DOCX) [file pone.0337757.s001.docx]

**S1 Appendix. Calculating Cigar-Smoking-Attributable Fractions in Healthcare Expenditures and Annual Healthcare Expenditure Estimates**

The following conceptual diagram illustrates the data linkage approach and study design used to estimate annual healthcare expenses, which are the primary outcome of this study, incurred by cigar smokers and non-smokers across public and private healthcare payer groups. Note that individuals in the U.S. may have multiple healthcare payers during a single year.


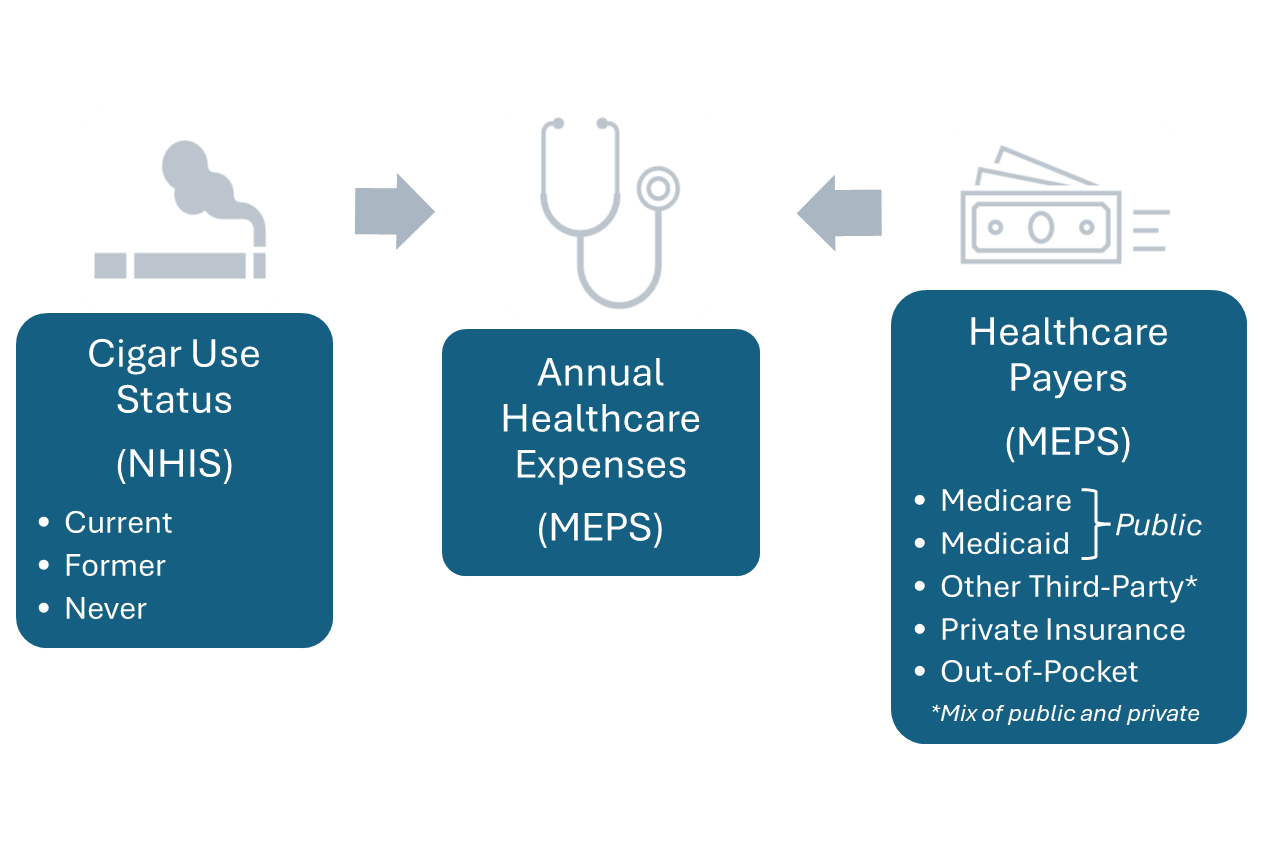
*Note: Controls for demographic variables and risky behaviors come from NHIS.*

The models used in the two- and four-part model approach to calculate the cigar-smoking-attributable fractions in healthcare expenditures are described below.

**Two-part model**

Part 1: performed a logit model to estimate the probability of having positive healthcare expenditures for respondent i at time t

${DHCExp}_{it}=\alpha_{t}+\beta{1Cigar SmokingStatus}_{it}+\delta{SocialDemo}_{it}+{\pi HlthBeh}_{it}+\varepsilon$

Where:

${DHCExp}_{it}:$ An indicator for positive healthcare expenditures for an individual respondent i at year t

Part 2: conducted a generalized linear model (with a log link and gamma distribution) to estimate annual attributable expenditures conditioned upon having positive healthcare expenditures

${HCExp}_{it}=\alpha_{t}^{'}+{\beta1}^{'}{Cigar SmokingStatus}_{it}+{SocialDemo}_{it}+{\pi^{'}HlthBeh}_{it}+\varepsilon^{'}$

Where:

${HCExp}_{it}$: Annual attributable expenditures conditioned on having positive healthcare expenditures for individual respondent i at year t

In both models:

$\beta{1Cigar SmokingStatus}_{it}:$key independent variables in the model, which is cigar-smoking status

$\delta{SocialDemo}_{it}$*:* Demographics (age group, sex, race/ethnicity, education, and marital status)

${\pi HlthBeh}_{it}$: Control variables for cigarette-smoking status, alcohol consumption, and past 12-month influenza vaccine

Estimates from both parts of the model were used to predict the proportion of healthcare expenditures for the overall study sample that could be reduced if all current and former cigar smokers had never smoked cigars. Predicted annual cigar-smoking-attributable healthcare expenditures were estimated by calculating the difference between predicted individual annual healthcare expenditures for current or former cigar smokers and their predicted individual annual expenditures if they had never smoked cigars in their lifetime (the counterfactual). The attributable fraction was then estimated by dividing the total cigar-smoking-attributable healthcare expenditures by the total predicted expenditures for the entire population.

**Four-part model**

Conducting a four-part regression model for total healthcare expenditures involves two additional steps because it separates inpatient healthcare expenditures from other healthcare expenditures:

Part 1: used a logit model to regress a binary indicator of any positive healthcare expenditures on independent indicators for demographics and smoking status for individual i in year t:

${DExp}_{it}=\alpha_{t}+\beta{1Cigar SmokingStatus}_{it}+\delta{SocialDemo}_{it}+\varepsilon$

Part 2: ran a generalized linear model with the positive healthcare expenditures for individual i and year t, if individual had no inpatient care expenditures but had positive other expenditures:

${ExpNIP}_{it}=\alpha_{t}^{'}+{\beta1}^{'}{Cigar SmokingStatus}_{it}+{SocialDemo}_{it}+\varepsilon^{'}$ if ${DC}_{it}=0$ & ${DExp}_{it}=1$

Part 3: used a logit model to regress a binary indicator of any positive inpatient care expenditures on independent indicators for demographics and smoking status (given positive annual positive expenditure) for individual i in year t:

${DIP}_{it}=\alpha_{t}+\beta{1Cigar SmokingStatus}_{it}+\delta{SocialDemo}_{it}+\varepsilon if {DExp}_{it}=1$

Part 4: ran a generalized linear model with the healthcare expenditures for individual i and year t, if individual had positive inpatient care expenditures:

${ExpIP}_{it}=\alpha_{t}^{'}+{\beta1}^{'}{Cigar SmokingStatus}_{it}+{SocialDemo}_{it}+\varepsilon^{'}$ if ${DC}_{it}=1$

The estimates were predicted (post-estimation) from the four models and the predicted estimates were then combined to apply the formula:

$$Exp=Pr(C*ExpIP+\left[ 1-C \right]*ExpNIP)$$

where $Exp$ represents predicted annual expenditures; Pr represents the predicted probability of having any positive healthcare expenditures estimated from part 1; $C$ represents the conditional probability of positive inpatient expenditures, given positive expenditure, estimated from part 2; $ExpIP$ represents predicted inpatient care expenditures, given positive inpatient expenditures, estimated from part 4; and $ExpNIP$ represents predicted healthcare expenditures, given positive expenditures but no inpatient expenditures, estimated from part 3.

**Copas tests**

Because healthcare expenditures present a highly-skewed distribution with many zeros, Copas tests were used to evaluate over-fitting and misspecification among the two- and four-part models [18, 34]. Copas tests showed that the four-part model slope was closer to 1 than that of the two-part model, suggesting the four-part model was a better fit. Additionally, the four-part model Copas test results had a range of values for the intercept that included zero, whereas the range of values for the two-part model Copas intercept did not include zero, suggesting the four-part model was a better fit for this study.

**Cigar-smoking-attributable healthcare expenditures**

National Health Expenditures Accounts–Personal Health Care (NHEA-PHC) expenditures are reported by the Centers for Medicare & Medicaid Services (CMS) among all individuals. For this study, expenditures for individuals aged 25 years and older were subtracted from the NHE-PHC expenditures, using Medical Expenditure Panel Survey (MEPS) data to estimate the fraction of MEPS expenditures for individuals aged 0–24 years:

1. Used MEPS age variable (ages 0–85 and older) to identify participants who were younger than 25 in each MEPS year.
2. Used MEPS annual analytic person weight variable (perwtYYf) to calculate the population aged 0–24 years. The MEPS population aged 0–24 years population was compared to the American Community Survey (ACS) U.S. aged 0–24 years population [1] to confirm the accuracy of MEPS numbers.
3. Calculated the weighted proportion for this age group (0–24 years) in each MEPS year, then multiplied this proportion by the NHE expenditures of the relevant MEPS year to estimate the proportion of NHE expenditures for 0–24-year-old individuals.
4. Subtracted the NHE expenditures of 0–24-year-olds from the overall NHE expenditures to estimate the NHE expenditures for adults aged 25 years and older.
5. Adjusted the 2001–2017 NHE expenditures to real 2018 dollars using the PHC Price Index multiplier and then took the average for all the study years.
6. To predict the healthcare expenditures attributable to cigar-smoking, the calculated attributable fraction was multiplied by the average annual NHE expenditures for adults aged 25 and older. The predicted expenditures were then bootstrapped to estimate the confidence intervals.

# **References**

1. Age and Sex. American Community Survey, ACS 1-Year Estimates Subject Tables, Table S0101. [Internet]. Washington (DC): U.S. Census Bureau, U.S. Department of Commerce. 2023 [cited 2025 May 20]. Available from: <https://data.census.gov/table/ACSST1Y2023.S0101?q=S01&d=ACS+1-Year+Estimates+Subject+Tables>
