# Supplementary material for: Annual U.S. healthcare expenditures attributable to cigar smoking between 2001 and 2018, overall and by payer
Source: PLoS One. 2025 Dec 1;20(12):e0337757. doi: 10.1371/journal.pone.0337757 (PMC12668525; doi:10.1371/journal.pone.0337757)
Supplement: S2 Appendix — (DOCX) [file pone.0337757.s002.docx]

**S2 Appendix. NHIS-MEPS Individual-Level Weight Adjustment**

Each MEPS full year of consolidated publicly available data includes individual-level weight that is assigned to survey participants who were “in scope” sometime during the survey year and responded for the full period in which they were in scope.

The individual-level weight was developed following these steps:

[1] Separate individual-level panel weight was created for each panel in the MEPS full-year data.

[2] Each panel weight was adjusted for nonresponse and calibrated to independent calibration totals (raked to Current Population Survey [CPS] population estimates for five demographic variables: age group, sex, race/ethnicity, poverty status, and regions).

[3] Each panel weight was then multiplied by a correction factor that reflected the relative sample size of each panel compared to the sample size for the two panels combined.

[4] The panel weights were then combined to become the final full-year person level weight, which was then raked again using the same CPS demographic variables in addition to the poverty status population total.

More information about the development of the MEPS individual-level weights can be found in the corresponding MEPS Full Year Consolidated data file document for MEPS Consolidated Data File, available from the U.S. Department of Health and Human Services Agency for Healthcare Research and Quality (AHRQ) website (<https://meps.ahrq.gov/mepsweb/>).

To conduct the analysis for this study, for each MEPS full year of consolidated data, we included in the analysis adults from MEPS panel with cigar use information, who were in scope at some time of the MEPS survey year and completed National Health Interview Survey (NHIS) survey (their MEPS data can be linked to the corresponding NHIS data). We adjusted the individual-level weight of the eligible respondents to account for the exclusion of MEPS panel respondents who did not have cigar use information collected, or who had cigar use information but did not link to NHIS data. This weight adjustment allowed for the respondents’ total weight to be equal to the national population total for the same MEPS survey year.

For each MEPS panel that we utilized for this analysis, we conducted the following weight adjustment steps:

[1] We summed the individual-level weight for all in-scope participants who were in MEPS sometime during the survey year, overall and by demographics and individual-level characteristics that were used in calculating the annual MEPS weights (age group, sex, race/ethnicity, and poverty level).

[2] To adjust for nonresponse, we calibrated the annual individual-level MEPS weight of the in-scope respondents to the population totals calculated in step #1. To implement the calibration, we used SUDAAN command “Proc WTADJUST.”

[3] To determine the need for weight trimming, we compare the Unequal Weighted Effect (UWE) for MEPS annual person-level weight to the UWE for the adjusted weight calculated in step #2. UWE changes that were < 2.5 were considered minor and acceptable, suggesting no trimming would be needed. It is worth noting that weight trimming was part of generating the original annual MEPS weights, and since nonresponse is mostly missing at random, no adjustments were added to the variance of the weight.

[i] The UWEs were < 2.5 for all MEPS years. Thus, we did not conduct any trimming.

[4] After adjusting the person-level weight in each MEPS year, we stacked NHIS-MEPS for all years of interest to the study and divided the raked weight from step #2 by the number of MEPS years (panels) involved in the study so the weights could provide average population total for the stacked MEPS years.
