# Supplementary material for: Annual U.S. healthcare expenditures attributable to cigar smoking between 2001 and 2018, overall and by payer
Source: PLoS One. 2025 Dec 1;20(12):e0337757. doi: 10.1371/journal.pone.0337757 (PMC12668525; doi:10.1371/journal.pone.0337757)
Supplement: S3 Appendix — (DOCX) [file pone.0337757.s003.docx]

# S3 Appendix. Additional Results

Table A. Variable Definitions and Data Sources.

| Analytic Variable Name | Variable Type | Data Source | Variable Definition |
| --- | --- | --- | --- |
| Age | Categorical: 25–44,  45–64, 65–74, and 75+ | NHIS | Age of respondent, reconstructed based on both reported age and date of birth |
| Sex | Categorical: Male, and Female | NHIS | Respondent reported sex, male or female |
| Education | Categorical: < High school, High school graduate or GED, Some college or Associate degree, and College degree or above | NHIS | Respondent reported highest level of school completed |
| Race/ethnicity | Categorical: White, non-Hispanic, Black, non-Hispanic, Other, non-Hispanic, and Hispanic | NHIS | Respondent reported race and Hispanic ethnicity |
| Marital status | Categorical: Married/ cohabitating, Never married, and Divorced/ widowed | NHIS | Respondent reported marital status |
| Alcohol consumption | Categorical: Current drinker, Former drinker, Lifetime abstainer | NHIS | Respondent reported lifetime alcohol consumption of 12+ drinks, and any alcohol consumption during the past 12 months |
| Past 12-month influenza vaccine | Dichotomous: Yes = 1, No = 0 | NHIS | Respondent reported having received an influenza vaccine during the past 12 months |
| Ever cigar-smoking | Dichotomous: Yes = 1, No = 0 | NHIS | Respondent reported either being current or former user of large cigars, cigarillos, or little filtered cigars |
| 3-category cigar-smoking status | Categorical: Current cigar smokers, Former cigar smokers, and Never cigar smokers | NHIS | Respondent reported cigar smoking status for main analyses: Current cigar smoker (some days or every day), Former cigar smoker (ever in lifetime but not smoking cigars at all currently), and Lifetime never cigar smoker (no reported lifetime cigar use) |
| 4-category cigar-smoking status | Categorical: Current cigar smokers, Former regular cigar smokers, Former cigar experimenters, and Never cigar smokers | NHIS (every 5 years only through 2015) | Respondent reported cigar smoking status for cigar threshold sensitivity analysis: Current cigar smoker (smoked some days or every day at the time of interview), Former regular cigar smoker (smoked 50+ cigars in lifetime, but did not smoke cigars at all currently), Former cigar experimenter (smoked < 50 cigars in lifetime, but did not smoke cigars at all currently), and Lifetime never cigar smoker (no reported lifetime cigar use) |
| Ever cigarette-smoking status | Dichotomous: Yes=1, No=0 | NHIS | Respondent reported either being current or former cigarette smoker |
| Cigarette-smoking status | Categorical: Current cigarette smoker, Former smokers, and never smoker: | NHIS | Self-reported cigarette smoking status: Current cigarette smoker (smoked 100 cigarettes in lifetime and smoked cigarettes some days or every day at the time of interview), Former smoker (smoked 100 cigarettes in lifetime but did not smoke cigarettes at all currently), and never smoker (smoked < 100 cigarettes in lifetime) |
| Total expenditure | Continuous | MEPS | Individual-level healthcare expenditure during past 12 months from all sources |
| Medicare expenditure | Continuous | MEPS | Individual-level healthcare expenditure during past 12 months paid by Medicare |
| Medicaid expenditure | Continuous | MEPS | Individual-level healthcare expenditure during past 12 months paid by Medicaid |
| Private insurance expenditure | Continuous | MEPS | Individual-level healthcare expenditure during past 12 months paid by private insurance |
| Out-of-pocket expenditure | Continuous | MEPS | Individual-level healthcare expenditure during past 12 months paid by self/family |
| Other third-party expenditure | Continuous | MEPS | Individual-level healthcare expenditure during past 12 months paid by Department of Veterans Affairs/ CHAMPVA; TRICARE; other federal sources; other state and local sources; Worker’s Compensation; other public; and other unclassified sources |

Table B. Sample Definitions for Payer and Primary Payer Groups Used in the Four-Part Model.

| Payer | Cigar-Smoking-Attributable Fractions and Annual Healthcare Expenditures by Payer | Sensitivity Analysis of Cigar-Smoking-Attributable Fractions and Annual Healthcare Expenditures by Primary Payer |
| --- | --- | --- |
| Medicare | Individuals who ever had Medicare insurance in the past 12 months or had positive Medicare expenditures. | Individuals with [(Medicare expenditure/total expenditure) X 100] > 50% or [total expenditure = 0 and ever had Medicare insurance (in the past 12 months)] (excluding individuals with < 50% of annual expenditures from Medicare). |
| Medicaid | Individuals who ever had Medicaid insurance in the past 12 months or had positive Medicaid expenditures. | Individuals with [(Medicaid expenditure/total expenditure) X 100] > 50% or [total expenditure = 0 and ever had Medicaid insurance (in the past 12 months)] (excluding individuals with < 50% of annual expenditures from Medicaid). |
| Private | Individuals who ever had private insurance in the past 12 months or had positive private insurance expenditures. | Individuals with [(private insurance expenditure/total expenditure) X 100] > 50% or [total expenditure = 0 and ever had private insurance (in the past 12 months)] (excluding individuals with < 50% of annual expenditures from private insurance). |
| Out-of-pocket | Individuals who had out-of-pocket expenditure > 0 or total expenditure = 0. | Individuals with [(out-of-pocket expenditure/total expenditure) X 100] > 50% or total expenditure = 0 (regardless of their insurance coverage status in the past 12 months). |
| Other third-party | Individuals with "other third-party" expenditure > 0 or (total expenditure = 0 & reported ever had insurance coverage in MEPS survey year). Using "other third-party" expenditures as the outcome in the regression models. | Individuals with [(other third-party expenditure/total expenditure) X 100] > 50% or total expenditure = 0 & reported ever had insurance coverage in MEPS survey year. |

Table C. Sample Definitions and Percentages of Single and Multiple Insurance Payers, Overall and by Payer Group.

| Payer (n)^b^ | Single Payer Sample^a^ | | Multiple Payers Sample | |
| --- | --- | --- | --- | --- |
|  | Definition | % | Definition | % |
| Overall  (n = 47,418) | Total healthcare expenditure > 0 and paid by one insurance payer group. | 10.7 | Total healthcare expenditure > 0 and paid by more than one insurance payer group. | 89.3 |
| Medicare  (n = 15,368) | Ever had Medicare in past 12 months and Medicare expenditure > 0 and Medicare expenditure = total healthcare expenditure. | 0.6 | Ever had Medicare in past 12 months and Medicare expenditure > 0 and Medicare expenditure < total healthcare expenditure. | 99.4 |
| Medicaid  (n = 7,374) | Ever had Medicaid in past 12 months and Medicaid expenditure > 0 and Medicaid expenditure = total healthcare expenditure. | 10.5 | Ever had Medicaid in past 12 months and Medicaid expenditure > 0 and Medicaid expenditure < total healthcare expenditure. | 89.5 |
| Private  (n = 32,526)^c^ | [Ever had private insurance in past 12 months and private insurance expenditure > 0] or “other private insurance” > 0 and total private insurance, expenditure = total healthcare expenditure. | 2.6 | [Ever had private insurance in past 12 months and private insurance expenditure > 0] or “other private insurance” > 0 and total private insurance, expenditure < total healthcare expenditure. | 97.4 |
| Out-of-pocket  (n = 44,802)^d^ | Out-of-pocket expenditure > 0 and out-of-pocket expenditure = total healthcare expenditure. | 6.8 | Out-of-pocket expenditure > 0 and out-of-pocket expenditure < total healthcare expenditure. | 93.2 |
| Other third-party  (n = 8,196)^e^ | “Other third-party payers” expenditure > 0 and “other third-party payers” expenditure = total healthcare expenditure. | 3.9 | Ever had insurance coverage = 1 and “other third-party payers” expenditure > 0 and “other third-party payers” expenditure < total healthcare expenditure. | 96.1 |

Notes: Weighted estimates are for U.S. adults aged 25 years and older, National Health Interview Survey-linked Medical Expenditure Panel Survey 2001–2018.
^a^ By definitions of “single payer” and “multiple payers” categories, respondents with $0 healthcare expenditure were not included in the table.
^b^ Sample sizes, overall and for each payer group.
^c^ Individuals in the private insurance group also include respondents with “other private insurance” (n = 4,590).
^d^ Individuals in the out-of-pocket group include respondents who did not report any health insurance but have positive “out-of-pocket” expenditures and respondents with one or more health insurance and out-of-pocket copayments. The latter, however, would be considered having multiple payers under this definition.

^e^ “Single payer” under the “other payers” group includes individuals with “other payers” healthcare expenditures equal to “overall” healthcare expenditure, regardless of whether they ever had insurance in the past 12 months, including 318 individuals with only “other payers” expenditures, among them 125 who reported no other insurance. “Multiple payers” under the “other payers” group includes individuals who ever had insurance in the past 12 months and their healthcare expenditures were paid by multiple insurance payers.

Table D. Weighted Annual Estimates of Cigar-Smoking Status, NHIS-Linked MEPS 2001–2018.

|  | Weighted % (95% CI) | | |
| --- | --- | --- | --- |
| NHIS Year | Cigar-Smoking Status | | |
|  | Current | Former | Never |
| 2000 | 3.9% (3.3 – 4.5) | 14.8% (13.6 – 16.0) | 81.4% (79.9 – 82.7) |
| 2005 | 3.5% (2.9 – 4.3) | 25.7% (23.7 – 27.9) | 70.7% (68.5 – 72.9) |
| 2010 | 4.6% (3.8 – 5.5) | 33.4% (31.6 – 35.4) | 62.0% (60.0 – 64.0) |
| 2015 | 3.4% (2.8 – 4.1) | 24.7% (22.8 – 26.6) | 72.0% (70.0 – 73.9) |
| 2016 | 3.6% (3.1 – 4.3) | 27.0% (25.4 – 28.7) | 69.3% (67.6 – 71.0) |
| 2017 | 4.0% (3.3 – 4.8) | 27.7% (26.1 – 29.3) | 68.3% (66.7 – 69.9) |

U.S. adults aged 25 years and older, National Health Interview Survey-linked Medical Expenditure Panel Survey 2001–2018.

Table E. Weighted Descriptive Statistics Overall and by Payer.

| Characteristics | Overall | Medicare^a^ | Medicaid^a^ | Private^a^ | Out-of-Pocket^a^ | Other Third-Party^a^ |
| --- | --- | --- | --- | --- | --- | --- |
|  | Unweighted | | | | | |
|  | n = 53,733 | n = 16,725 | n = 8,915 | n = 37,065 | n = 51,298 | n = 13,564 |
|  | Weighted % (95% CI)^b^ | | | | | |
| Age group (years) | | | | | | |
| 25–44 | 42.5 (42.0, 43.0) | 2.9 (2.6, 3.2) | 41.6 (40.3, 42.9) | 43.4 (42.8, 44.0) | 41.8 (41.2, 42.3) | 39.7 (38.7, 40.8) |
| 45–64 | 38.0 (37.5, 38.5) | 21.0 (20.2, 21.7) | 36.8 (35.6, 38.1) | 39.2 (38.6, 39.8) | 38.4 (37.8, 38.9) | 36.7 (35.7, 37.7) |
| 65–74 | 10.8 (10.5, 11.1) | 42.0 (41.1, 42.9) | 12.0 (11.2, 12.9) | 9.6 (9.3, 10.0) | 11.0 (10.7, 11.3) | 12.4 (11.8, 13.1) |
| 75+ | 8.7 (8.5, 9.0) | 34.2 (33.3, 35.1) | 9.5 (8.8, 10.3) | 7.8 (7.5, 8.1) | 8.9 (8.6, 9.2) | 11.1 (10.5, 11.8) |
| Sex | | | | | | |
| Male | 48.0 (47.5, 48.5) | 44.6 (43.7, 45.6) | 36.7 (35.4, 38.0) | 47.8 (47.2, 48.4) | 47.7 (47.2, 48.2) | 57.5 (56.5, 58.5) |
| Female | 52.0 (51.5, 52.5) | 55.4 (54.4, 56.3) | 63.3 (62.0, 64.6) | 52.2 (51.6, 52.8) | 52.3 (51.8, 52.8) | 42.5 (41.5, 43.5) |
| Education | | | | | | |
| < High school | 12.5 (12.2, 12.8) | 20.3 (19.6, 21.0) | 30.3 (29.1, 31.4) | 8.1 (7.8, 8.4) | 12.4 (12.1, 12.7) | 14.6 (13.9, 15.2) |
| High school graduate or GED | 23.9 (23.4, 24.3) | 29.1 (28.2, 29.9) | 30.8 (29.6, 32.0) | 21.9 (21.4, 22.4) | 23.9 (23.4, 24.3) | 27.3 (26.4, 28.2) |
| Some college or associate degree | 29.3 (28.8, 29.8) | 27.1 (26.3, 27.9) | 28.1 (26.9, 29.3) | 29.8 (29.3, 30.4) | 29.3 (28.8, 29.8) | 31.4 (30.4, 32.4) |
| College degree or above | 34.3 (33.8, 34.8) | 23.6 (22.8, 24.4) | 10.8 (10.0, 11.8) | 40.2 (39.6, 40.8) | 34.5 (34.0, 35.0) | 26.7 (25.8, 27.7) |
| Race/ethnicity | | | | | | |
| White,  non-Hispanic | 69.1 (68.7, 69.6) | 77.8 (77.2, 78.5) | 48.6 (47.3, 49.9) | 73.8 (73.3, 74.2) | 69.7 (69.3, 70.2) | 65.2 (64.3, 66.2) |
| Black,  non-Hispanic | 11.4 (11.1, 11.6) | 10.2 (9.8, 10.7) | 22.3 (21.4, 23.3) | 9.8 (9.5, 10.1) | 11.1 (10.9, 11.4) | 13.6 (13.0, 14.2) |
| Hispanic | 12.6 (12.3, 13.0) | 7.5 (7.1, 8.0) | 20.5 (19.6, 21.5) | 9.5 (9.2, 9.8) | 12.4 (12.1, 12.7) | 14.0 (13.4, 14.6) |
| Other,  non-Hispanic | 6.8 (6.6, 7.1) | 4.4 (4.1, 4.8) | 8.5 (7.8, 9.4) | 6.9 (6.6, 7.2) | 6.7 (6.4, 7.0) | 7.2 (6.6, 7.7) |
| Marital status | | | | | | |
| Married/ cohabitating | 53.8 (53.3, 54.3) | 44.3 (43.4, 45.2) | 32.5 (31.3, 33.8) | 57.7 (57.1, 58.4) | 53.9 (53.4, 54.5) | 52.7 (51.7, 53.8) |
| Never married | 18.4 (18, 18.9) | 8.5 (8.0, 9.0) | 29.0 (27.8, 30.2) | 17.2 (16.7, 17.7) | 18.1 (17.7, 18.6) | 17.5 (16.7, 18.4) |
| Divorced/ widowed | 27.8 (27.3, 28.2) | 47.2 (46.3, 48.2) | 38.5 (37.3, 39.8) | 25.0 (24.5, 25.6) | 27.9 (27.5, 28.4) | 29.7 (28.8, 30.7) |
| Alcohol consumption | | | | | | |
| Current drinker | 67.6 (67.1, 68.1) | 50.7 (49.7, 51.6) | 47.7 (46.3, 49.0) | 71.6 (71.0, 72.1) | 67.7 (67.3, 68.2) | 65.1 (64.1, 66.1) |
| Former drinker | 14.7 (14.4, 15.1) | 24.4 (23.6, 25.2) | 22.9 (21.8, 24.1) | 12.9 (12.6, 13.3) | 14.7 (14.4, 15.1) | 17.3 (16.5, 18.1) |
| Lifetime abstainer | 17.7 (17.3, 18.0) | 25.0 (24.2, 25.8) | 29.4 (28.3, 30.6) | 15.5 (15.1, 15.9) | 17.5 (17.2, 17.9) | 17.6 (16.9, 18.4) |
| **Past-12-month influenza vaccine** |  |  |  |  |  |  |
| Yes | 36.9 (36.4, 37.4) | 37.6 (36.7, 38.5) | 36.3 (35.0, 37.6) | 38.3 (37.7, 38.8) | 37.1 (36.6, 37.4) | 38.5 (37.5, 39.5) |
| No | 63.1 (62.6, 63.6) | 62.4 (61.5, 63.3) | 63.7 (62.4, 65.0) | 61.7 (61.2, 62.3) | 62.9 (62.4, 63.4) | 61.5 (60.5, 62.5) |

Notes: Weighted estimates are for U.S. adults aged 25 years and older, National Health Interview Survey-linked Medical Expenditure Panel Survey 2001–2018. Cigar and cigarette use results are presented in Table 1.

^a^ The respondents within each payer are not mutually exclusive, because respondents can have multiple payers within a calendar year.

^b^ The proportions of the categories of each variable are based on the number of respondents with non-missing values for that variable in the sample.

Table F. Weighted Descriptive Statistics Overall and by Healthcare Payer Mix.

| Characteristics | Overall | Zero Expenditure^a^ | Single Payer (Any)^a, b^ | Multiple Payers^a, c^ |
| --- | --- | --- | --- | --- |
|  | Unweighted | | | |
|  | n = 53,733 | n = 6,315 | n = 5,076 | n = 42,342 |
|  | Weighted % (95% CI)^d^ | | | |
| Age group (years) |  |  |  |  |
| 25–44 | 42.5 (42.0, 43.0) | 65.6 (64.1, 67) | 61.1 (59.4, 62.8) | 37.3 (36.8, 37.9) |
| 45–64 | 38.0 (37.5, 38.5) | 29.6 (28.2, 31) | 34.4 (32.8, 36.1) | 39.5 (38.9, 40.1) |
| 65–74 | 10.8 (10.5, 11.1) | 3.0 (2.6, 3.5) | 2.8 (2.3, 3.3) | 12.7 (12.4, 13.1) |
| 75+ | 8.7 (8.5, 9.0) | 1.9 (1.5, 2.4) | 1.7 (1.3, 2.1) | 10.4 (10.1, 10.8) |
| Sex |  |  |  |  |
| Male | 48.0 (47.5, 48.5) | 67.0 (65.6, 68.4) | 56.3 (54.6, 58.0) | 44.5 (43.9, 45.1) |
| Female | 52.0 (51.5, 52.5) | 33.0 (31.6, 34.4) | 43.7 (42.0, 45.4) | 55.5 (54.9, 56.1) |
| Education |  |  |  |  |
| < High school | 12.5 (12.2, 12.8) | 20.1 (19.0, 21.3) | 14.8 (13.8, 15.9) | 11.3 (10.9, 11.6) |
| High school graduate or GED | 23.9 (23.4, 24.3) | 29.5 (28.1, 30.9) | 26.6 (25.1, 28.2) | 22.8 (22.4, 23.3) |
| Some college or associate degree | 29.3 (28.8, 29.8) | 27.7 (26.3, 29.2) | 29.6 (28.0, 31.2) | 29.5 (28.9, 30.0) |
| College degree or above | 34.3 (33.8, 34.8) | 22.7 (21.3, 24.2) | 29.0 (27.4, 30.7) | 36.4 (35.9, 37.0) |
| Race/ethnicity |  |  |  |  |
| White, non-Hispanic | 69.1 (68.7, 69.6) | 50.4 (48.8, 51.9) | 58.1 (56.4, 59.7) | 72.9 (72.4, 73.3) |
| Black, non-Hispanic | 11.4 (11.1, 11.6) | 15.1 (14.2, 16.1) | 12.8 (11.8, 13.8) | 10.7 (10.4, 11.0) |
| Hispanic | 12.6 (12.3, 13.0) | 25.8 (24.5, 27.0) | 20.6 (19.3, 21.9) | 10.0 (9.7, 10.3) |
| Other, non-Hispanic | 6.8 (6.6, 7.1) | 8.7 (7.9, 9.7) | 8.6 (7.7, 9.6) | 6.4 (6.1, 6.7) |
| Marital status |  |  |  |  |
| Married/ cohabitating | 53.8 (53.3, 54.3) | 53.8 (52.3, 55.4) | 50.5 (48.8, 52.3) | 54.2 (53.6, 54.8) |
| Never married | 18.4 (18, 18.9) | 25.3 (23.9, 26.7) | 25.7 (24.1, 27.4) | 16.7 (16.3, 17.2) |
| Divorced/ widowed | 27.8 (27.3, 28.2) | 20.9 (19.7, 22.2) | 23.8 (22.3, 25.3) | 29.1 (28.6, 29.6) |
| Alcohol consumption |  |  |  |  |
| Current drinker | 67.6 (67.1, 68.1) | 68.6 (67.1, 70.0) | 69.3 (67.7, 70.8) | 67.3 (66.8, 67.8) |
| Former drinker | 14.7 (14.4, 15.1) | 11.4 (10.5, 12.5) | 12.2 (11.1, 13.4) | 15.4 (15.0, 15.8) |
| Lifetime abstainer | 17.7 (17.3, 18.0) | 20.0 (18.8, 21.2) | 18.5 (17.3, 19.8) | 17.3 (16.9, 17.7) |
| **Past-12-month influenza vaccine** |  |  |  |  |
| Yes | 36.9 (36.4, 37.4) | 15.4 (14.3, 16.5) | 19.7 (18.3, 21.2) | 41.6 (41.1, 42.2) |
| No | 63.1 (62.6, 63.6) | 84.6 (83.5, 85.7) | 80.3 (78.9, 81.7) | 58.4 (57.8, 58.9) |

Notes: Weighted estimates are for U.S. adults aged 25 years and older, National Health Interview Survey-linked Medical Expenditure Panel Survey 2001–2018. Cigar and cigarette use results are presented in Table 2.

^a^ The respondents within each healthcare expenditure and payer mix are mutually exclusive.

^b^ “Single payer” is defined as having healthcare expenditure and have only one of the five primary payer groups: Medicare, Medicaid, private insurance, out-of-pocket, or other payers within a calendar year.

^c^ “Multiple payers” is defined as having healthcare expenditure and have at least two of the five primary payer groups in a calendar year.

^d^ The proportions of the categories of each variable are based on the number of respondents with non-missing values for that variable in the sample.

Table G. Weighted Descriptive Statistics Overall and by Primary Payer.

| Characteristics | Overall | Medicare | Medicaid | Private | Out-of-Pocket | Other Third-Party |
| --- | --- | --- | --- | --- | --- | --- |
|  | Unweighted N | | | | | |
|  | n = 53,733 | n = 9,963 | n = 4,952 | n = 21,083 | n = 16,371 | n = 6,735 |
|  | Weighted % (95% CI)) | | | | | |
| Ever cigar-smoking status | | | | | | |
| Ever | 29.1 (28.7, 29.6) | 24.8 (23.7, 25.9) | 23.4 (21.9, 24.9) | 30.2 (29.5, 31.0) | 30.1 (29.2, 31.0) | 33.9 (32.4, 35.3) |
| Never | 70.9 (70.4, 71.3) | 75.2 (74.1, 76.3) | 76.6 (75.1, 78.1) | 69.8 (69.0, 70.5) | 69.9 (69.0, 70.8) | 66.1 (64.7, 67.6) |
| Current cigar-smoking status | | | | | | |
| Current | 3.8 (3.6, 4.1) | 2.3 (2.0, 2.8) | 3.2 (2.6, 3.8) | 4.1 (3.7, 4.4) | 4.6 (4.2, 5.0) | 5.4 (4.6, 6.2) |
| Former | 25.3 (24.8, 25.8) | 22.4 (21.4, 23.5) | 20.2 (18.8, 21.7) | 26.2 (25.4, 26.9) | 25.5 (24.7, 26.4) | 28.5 (27.1, 29.9) |
| Never | 70.9 (70.4, 71.3) | 75.2 (74.1, 76.3) | 76.6 (75.1, 78.1) | 69.8 (69, 70.5) | 69.9 (69.0, 70.8) | 66.1 (64.7, 67.6) |
| Ever cigarette-smoking status | | | | | | |
| Ever | 43.0 (42.5, 43.6) | 50.3 (49.1, 51.5) | 51.2 (49.4, 52.9) | 38.0 (37.3, 38.8) | 42.9 (41.9, 43.8) | 46.4 (44.9, 47.9) |
| Never | 57.0 (56.4, 57.5) | 49.7 (48.5, 50.9) | 48.8 (47.1, 50.6) | 62.0 (61.2, 62.7) | 57.1 (56.2, 58.1) | 53.6 (52.1, 55.1) |
| Current cigarette-smoking status | | | | | | |
| Current | 19.2 (18.8, 19.6) | 13.3 (12.6, 14.2) | 33.6 (32, 35.3) | 17.0 (16.4, 17.7) | 23.3 (22.5, 24.1) | 25.4 (24.1, 26.7) |
| Former | 23.8 (23.4, 24.3) | 37.0 (35.8, 38.2) | 17.5 (16.2, 19.0) | 21.0 (20.3, 21.7) | 19.5 (18.8, 20.3) | 21.0 (19.8, 22.3) |
| Never | 57.0 (56.4, 57.5) | 49.7 (48.5, 50.9) | 48.8 (47.1, 50.6) | 62.0 (61.2, 62.8) | 57.1 (56.2, 58.1) | 53.6 (52.1, 55.1) |
| Age group (years) | | | | | | |
| 25–44 | 42.5 (42.0, 43.0) | 3.6 (3.1, 4.1) | 53.5 (51.7, 55.3) | 52.7 (51.9, 53.5) | 52.7 (51.8, 53.7) | 53.2 (51.7, 54.7) |
| 45–64 | 38.0 (37.5, 38.5) | 18.9 (18.0, 19.8) | 38.8 (37.1, 40.5) | 43.6 (42.8, 44.4) | 36.8 (35.9, 37.8) | 34.8 (33.4, 36.2) |
| 65–74 | 10.8 (10.5, 11.1) | 40.9 (39.7, 42.1) | 4.0 (3.4, 4.7) | 2.5 (2.3, 2.7) | 6.0 (5.7, 6.5) | 7.4 (6.7, 8.2) |
| 75+ | 8.7 (8.5, 9.0) | 36.7 (35.5, 37.8) | 3.7 (3.0, 4.5) | 1.2 (1.0, 1.4) | 4.4 (4.1, 4.8) | 4.6 (4.0, 5.3) |
| Sex | | | | | | |
| Male | 48.0 (47.5, 48.5) | 42.5 (41.3, 43.7) | 35.6 (33.8, 37.4) | 49.2 (48.4, 50.0) | 54.3 (53.3, 55.2) | 64.1 (62.7, 65.5) |
| Female | 52.0 (51.5, 52.5) | 57.5 (56.3, 58.7) | 64.4 (62.6, 66.2) | 50.8 (50.0, 51.6) | 45.7 (44.8, 46.7) | 35.9 (34.5, 37.3) |
| Education | | | | | | |
| < High school | 12.5 (12.2, 12.8) | 21.9 (20.9, 22.8) | 29.9 (28.4, 31.4) | 6.1 (5.8, 6.5) | 14.3 (13.7, 14.9) | 14.7 (13.8, 15.6) |
| High school graduate or GED | 23.9 (23.4, 24.3) | 29.3 (28.2, 30.4) | 31.3 (29.7, 33.0) | 20.1 (19.5, 20.7) | 25.7 (24.9, 26.5) | 28.8 (27.4, 30.1) |
| Some college or associate degree | 29.3 (28.8, 29.8) | 26.9 (25.9, 28.1) | 29.1 (27.4, 30.7) | 30.1 (29.4, 30.9) | 28.0 (27.1, 28.9) | 32.0 (30.6, 33.5) |
| College degree or above | 34.3 (33.8, 34.8) | 21.9 (20.8, 22.9) | 9.7 (8.7, 10.9) | 43.7 (42.9, 44.5) | 32.0 (31.1, 33.0) | 24.5 (23.2, 25.9) |
| Race/ethnicity | | | | | | |
| White,  non-Hispanic | 69.1 (68.7, 69.6) | 75.9 (75.0, 76.8) | 46.4 (44.7, 48.2) | 70.9 (70.2, 71.6) | 64.4 (63.5, 65.3) | 56.9 (55.4, 58.3) |
| Black,  non-Hispanic | 11.4 (11.1, 11.6) | 11.2 (10.6, 11.8) | 23.3 (21.9, 24.6) | 10.6 (10.2, 11.0) | 10.9 (10.4, 11.4) | 16.5 (15.5, 17.5) |
| Hispanic | 12.6 (12.3, 13.0) | 8.5 (8.0, 9.1) | 21.6 (20.4, 22.9) | 10.6 (10.2, 11.1) | 17.4 (16.8, 18.1) | 17.6 (16.6, 18.7) |
| Other,  non-Hispanic | 6.8 (6.6, 7.1) | 4.3 (3.9, 4.8) | 8.7 (7.7, 9.9) | 7.9 (7.4, 8.3) | 7.3 (6.8, 7.8) | 9.0 (8.2, 9.9) |
| Marital status | | | | | | |
| Married/ cohabitating | 53.8 (53.3, 54.3) | 41.1 (39.9, 42.3) | 36.1 (34.4, 37.9) | 61.6 (60.8, 62.4) | 53.0 (52, 53.9) | 54.3 (52.8, 55.8) |
| Never married | 18.4 (18, 18.9) | 9.5 (8.8, 10.2) | 32.0 (30.4, 33.7) | 18.4 (17.7, 19.0) | 22.6 (21.7, 23.4) | 20.8 (19.6, 22.1) |
| Divorced/ widowed | 27.8 (27.3, 28.2) | 49.4 (48.2, 50.6) | 31.9 (30.3, 33.5) | 20.1 (19.4, 20.7) | 24.5 (23.6, 25.3) | 24.9 (23.6, 26.2) |
| Alcohol consumption | | | | | | |
| Current drinker | 67.6 (67.1, 68.1) | 48.5 (47.3, 49.8) | 50.8 (49.0, 52.6) | 75.1 (74.4, 75.8) | 70.6 (69.8, 71.4) | 66.9 (65.5, 68.2) |
| Former drinker | 14.7 (14.4, 15.1) | 25.4 (24.3, 26.4) | 21.5 (20.0, 23.1) | 10.8 (10.4, 11.3) | 11.7 (11.1, 12.3) | 14.6 (13.6, 15.7) |
| Lifetime abstainer | 17.7 (17.3, 18.0) | 26.1 (25.1, 27.2) | 27.7 (26.2, 29.2) | 14.0 (13.5, 14.6) | 17.7 (17, 18.4) | 18.5 (17.4, 19.6) |
| **Past-12-month influenza vaccine** |  |  |  |  |  |  |
| Yes | 36.9 (36.4, 37.4) | 37.4 (36.3, 38.6) | 29.1 (27.5, 30.8) | 32.8 (32.1, 33.6) | 24.1 (23.2, 24.9) | 28.3 (27.0, 29.7) |
| No | 63.1 (62.6, 63.6) | 62.6 (61.4, 63.7) | 70.9 (69.2, 72.5) | 67.2 (66.4, 67.9) | 75.9 (75.1, 76.8) | 71.7 (70.3, 73.0) |

Note: Weighted estimates for U.S. adults aged 25 years and older, National Health Interview Survey-linked Medical Expenditure Panel Survey 2001–2018.

Table H. Cigar-Smoking-Attributable Fractions Estimated from the Two-Part Model and Annual Healthcare Expenditures, Overall and by Payer.

| Cigar-Smoking Status by Payer | Weighted % (95% CI) | |
| --- | --- | --- |
|  | Percent Attributable Fraction | NHEA 2001–2018 $ billions^a^ |
| **Total expenditure (n = 52,791)^b^** |  | |
| Ever | **2.2 (0.5 – 3.8)** | **36.2 (8.7 – 63.7)** |
| Current | 0.2 (-0.2 – 0.5) | — |
| Former | **2.0 (0.4 – 3.6)** | **33.2 (6.7 – 59.6)** |
| **Medicare (n = 16,375)** |  |  |
| Ever | -1.8 (-4.5 – 0.90) | — |
| Current | -0.1 (-0.1 – 0.50) | — |
| Former | -1.7 (-4.2 – 0.80) | — |
| **Medicaid (n = 8,681)** |  |  |
| Ever | 1.9 (-2.4 – 6.3) | — |
| Current | 0.0 (-1.3 – 1.3) | — |
| Former | 1.9 (-2.2 – 5.9) | — |
| **Private (n = 36,549)** |  |  |
| Ever | **2.4 (0.0 – 4.8)** | **13.4 (0.05 – 26.7)** |
| Current | 0.1 (-0.5 – 0.6) | — |
| Former | **2.3 (0.1 – 4.6)** | **13.3 (0.4 – 25.7)** |
| **Out-of-pocket (n = 50,411)** |  |  |
| Ever | 0.5 (-1.0 – 2.6) | — |
| Current | 0.1 (-0.3 – 0.4) | — |
| Former | 0.5 (-0.9 – 1.9) | — |
| **Other third-party (n = 13,299)^c^** |  |  |
| Ever | 0.0 (-3.5 – 3.4) | — |
| Current | 0.6 (-0.2 – 1.4) | — |
| Former | -0.6 (-3.8 – 2.6) | — |

Notes: Weighted estimates are for U.S. adults aged 25 years and older, National Health Interview Survey-linked Medical Expenditure Panel Survey 2001–2018. Boldface indicates statistical significance (*p* < 0.05) based on bootstrapped 95% CIs.
^a^ Dollar values were adjusted to 2018 dollars using the Personal Health Care Price Index.
^b^ Sample size excluded 942 individuals who had missing values for the regression covariates.
^c^ The National Health Expenditure Accounts (NHEA) definition for “other payers” includes other health insurance programs (Children's Health Insurance Program [Titles XIX and XXI], Department of Defense, and Department of Veterans Affairs) and other third-party payers (worksite healthcare, other private revenues, Indian Health Service, Workers’ Compensation, general assistance, maternal and child health, vocational rehabilitation, other federal programs, Substance Abuse and Mental Health Services Administration, other state and local programs, and school health).

Table I. Cigar Threshold Sensitivity Analysis: Cigar-Smoking-Attributable Fractions Estimated from the Four-Part Model and Annual Healthcare Expenditures, Overall, 2001–2016.

| Alternate Cigar-Smoking Status | Percent Attributable Fraction (95% CI) | NHEA 2001–2016 |
| --- | --- | --- |
|  |  | $ billions^a^ (95% CI) |
| **Total expenditure (n = 34,390)^b^** |  |  |
| Ever | **2.4 (0.8 - 4.0)** | **37.5 (12.6 - 62.4)** |
| Current | 0.3 (-0.0 - 0.6) | — |
| Former regular^c^ | **0.8 (0.1 - 1.6)** | **12.9 (1.7 – 24.1)** |
| Former experimenters^d^ | **1.3 (0.1 - 2.6)** | **20.5 (1.0 – 40.0)** |

Notes: Weighted estimates are for U.S. adults aged 25 years and older, National Health Interview Survey-linked Medical Expenditure Panel Survey 2001–2016. Boldface indicates statistical significance (*p* < 0.05) based on bootstrapped 95% CIs.

^a^ Dollar values were adjusted to 2016 dollars using the Personal Health Care-Price Index.

^b^ Sample size excluded 569 individuals who had missing values for the regression covariates.

^c^ Former regular cigar smokers are those who self-reported having used 50 cigars or more in their lifetime.

^d^ Former experimenters are those who self-reported having used less than 50 cigars in their lifetime.
